# Engineered gp120 immunogens that elicit VRC01-like antibodies by vaccination

**DOI:** 10.1186/1742-4690-9-S2-P6

**Published:** 2012-09-13

**Authors:** J Mata-Fink, M Hanson, B Kriegsman, D Irvine, K Wittrup

**Affiliations:** 1 Massachusetts Institute of Technology, Cambridge, MA, USA

## Background

One of the great challenges for an HIV vaccine is to elicit broadly neutralizing antibodies specific for conserved epitopes from which the virus cannot easily escape. The CD4 binding site is one such epitope against which several antibodies (e.g. b12, VRC01) have been isolated. In macaques infected with SHIV, passive immunization with these CD4-directed neutralizing antibodies fails to control the virus, but prophylactic administration is highly protective. Similarly, patients who generate neutralizing antibodies over the course of an HIV infection derive no clinical benefit from them, but eliciting such antibodies prophylactically by vaccination may prevent the virus from establishing its lethal foothold.

## Methods

Yeast surface display is a powerful method for rapidly engineering complex glycoproteins. We have developed a stripped core gp120 that presents a functional CD4 binding site when displayed on yeast, and an accompanying suite of tools with which to map conformational epitopes of neutralizing antibodies, design novel immunogens, and monitor the specificity of serum following immunization.

## Results

We map the epitopes of the anti-gp120 antibodies VRC01, b12, and b13, and uncover subtle energetic differences in their nearly-overlapping epitopes that are not obvious from existing crystal structures. With this information, we design novel gp120 immunogens that share the VRC01 epitope but whose other surface amino acids are highly diverse. When mice are immunized with these immunogens in a heterologous prime-boost format they elicit VRC01-competitive antibodies and not the competing immunodominant specificities seen with a single immunogen. The antisera are tested for their binding to a panel of gp120, and their neutralization potential is assayed.

## Conclusion

We have engineered a novel set of immunogens that elicit CD4 binding site-directed antibodies upon immunization. The yeast display tools may be used to design future immunogens.

